# Intravascular Large B-Cell Lymphoma Within the Appendix Presenting as Acute Abdomen: A Challenging Diagnosis for Hematologists

**DOI:** 10.4274/tjh.galenos.2020.2020.0289

**Published:** 2021-02-25

**Authors:** Semra Cemre Atalar, Olga Meltem Akay, Emre Osmanbaşoğlu, Helin Masyan, Orhun Çığ Taşkın, Burhan Ferhanoğlu

**Affiliations:** 1 Koç University Faculty of Medicine, İstanbul, Turkey; 2 Koç University Faculty of Medicine, Department of Hematology, İstanbul, Turkey; 3 Koç University Faculty of Medicine, Department of Pathology, İstanbul, Turkey

**Keywords:** Intravascular large B-cell lymphoma, Appendix

## To the Editor,

Intravascular large cell lymphoma (ILCL) is a rare subtype of non-Hodgkin lymphoma characterized by the proliferation of lymphoma cells within the lumen of small vessels. The clinical diagnosis of ILCL is challenging due to the absence of obvious lymphadenopathy or a detectable mass [1]. The disease is usually diagnosed postmortem or incidentally in patients with diverse signs and symptoms related to organ dysfunction caused by occlusion of blood vessels [2]. Herein, we report a case of intravascular large B-cell lymphoma (ILBCL) presenting as acute abdomen. The diagnosis was rendered following the histopathologic examination of an appendectomy specimen. To our knowledge, this is the first report of ILCL of B-cell origin with appendix involvement.

A 58-year-old male patient was referred to our hospital for investigation of the etiology of his pancytopenia. His laboratory tests demonstrated hemoglobin of 10.9 g/dL, white blood cell count of 800/µL, platelets of 48,000/µL, and lactate dehydrogenase of 625 U/L. Bone marrow biopsy revealed dyshematopoiesis, especially in megakaryocytic and granulocytic cell lines, with hypercellular bone marrow including patchy CD20+ B-cell infiltration in the peritrabecular zone. The intravascular pattern of infiltration was not prominent. Cytogenetic analysis revealed a complex karyotype, and FISH analysis showed trisomy 7 and 21. B-cell lymphoid infiltration in the bone marrow was identified as hematogone-like atypical B-cell proliferation, but further work-up including positron emission tomography/computed tomography (PET/CT) was planned for the differential diagnosis of lymphoproliferative disorders. On the 15^th^ day after his initial admission, the patient suddenly developed abdominal pain. Upon finding rebound tenderness and dilated appendix with a thickened, hyperenhancing wall on CT scan, the patient was sent to the general surgery department for consultation and underwent emergent laparoscopic appendectomy with suspicion of acute appendicitis. In gross examination, the appendix was within normal limits in size and shape.

**Figure 1:**
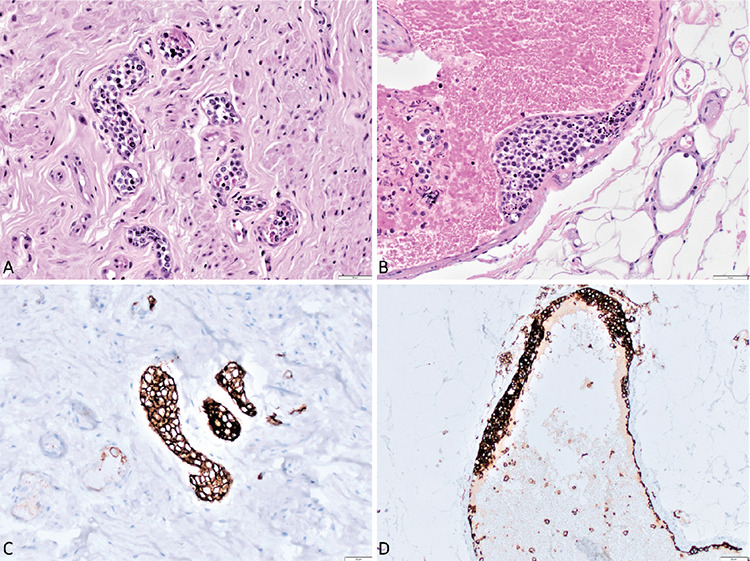
**A, B)** Infiltration of lymphoid cells in a small and large vessel in the periappendiceal area (H&E). **C, D)** CD20 immunohistochemistry, highlighting the B-cell phenotype of the neoplastic cells (CD20).

The histopathological investigation of the appendix yielded a final report of “high-proliferative, neoplastic B-cell infiltration in the vascular structures at the wall and serosa of the appendix” (Figure 1). Histological findings of acute appendicitis were absent. Meanwhile, the results of PET/CT performed prior to surgery were reported as “splenomegaly, hypermetabolic lesion on cecum at right hemipelvis (abscess?), intermediate hypermetabolism at axial skeleton and bilateral humerus-femur” after the operation. In light of these findings, the patient was diagnosed with intravascular large B-cell lymphoma and was given 6 cycles of R-CHOP and intrathecal methotrexate for central nervous system (CNS) prophylaxis. Bone marrow biopsy after 4 cycles of R-CHOP showed normal cytogenetics without any lymphocyte infiltration and complete metabolic remission was detected on interim PET/CT. Follow-up of the patient has been uneventful in the 12 months following the completion of treatment. 

Although rare cases of ILBCL of the gastrointestinal tract have been previously described [3,4,5,6], to our knowledge there have been no reports of ILCL of B-cell origin with appendix involvement in the English literature. The only reported case of ILCL involving the appendix was of T-cell origin, described by Malicki et al. [7] in 1999. In 2004, gastrointestinal involvement was reported at a rate of 8% (3 cases) in a series of 38 patients with ILCL by Ferreri et al. [8]. Since then, there have been limited case reports in which the gastrointestinal tract was the primary diagnostic site of ILBCL: three in the colon, two in the gastroduodenum, two in the duodenum, two in the ileum, and one in the stomach [3,5,6]. Treatment of ILBCL includes both systemic therapy with anthracycline-based regimens and therapy directed at the CNS, since CNS involvement is frequently seen [9]. Clinical outcomes for patients with ILBCL have improved in the rituximab (R) era. In the largest retrospective analysis to date, higher rates of overall and progression-free survival were reported in patients treated with R-chemotherapy compared to chemotherapy alone (66% and 56% vs. 46% and 27%, respectively) [10]. Our patient is the first to present with ILCL of B-cell origin, which was diagnosed as a result of an appendectomy. Early diagnosis in appendectomy and timely initiation of appropriate treatment allowed the successful response in this case. As a take-home message, late-onset acute appendicitis requires careful histological examination to exclude associated systemic diseases such as inflammatory bowel disease, pseudomyxoma, or lymphoma, as in the presented case.
